# A feasibility study on non-invasive and non-contact jugular venous pulse measurement using 60 GHz FMCW radar

**DOI:** 10.1098/rsos.242231

**Published:** 2025-06-25

**Authors:** Shatabdi Das, Hadi Afsharan, Girish Dwivedi, Coen Arrow, Omid Kavehei

**Affiliations:** ^1^ School of Biomedical Engineering, The University of Sydney, Sydney, New South Wales, Australia; ^2^ Advanced Clinical and Translational Cardiovascular Imaging, Harry Perkins Institute of Medical Research, Perth, Western Australia, Australia; ^3^ School of Medicine, The University of Western Australia, Perth, Western Australia, Australia; ^4^ School of Physics, The University of Western Australia, Perth, Western Australia, Australia; ^5^ The University of Sydney Nano Institute, Sydney, NSW 2006, Australia

**Keywords:** heart failure, noninvasive sensing, jugular venous pulse, mmWave sensing, signal processing, radio frequency

## Abstract

The jugular venous pulse (JVP) reflects right atrial pressure and provides diagnostic insight into cardiovascular and pulmonary health. However, reliable assessment remains difficult due to neck adiposity, anatomical variability and suboptimal positioning. Although central venous catheterization is the gold standard, its invasive nature restricts routine or long-term use. This study introduces a non-invasive method for JVP estimation using a 60 GHz frequency-modulated continuous wave (FMCW) radar. The system captures venous pulsations at the skin surface and applies eigenbeamforming to enhance signal-to-noise ratio and pulse clarity. Radar parameters were optimized for signal fidelity and validated through morphological comparison with simultaneously recorded photoplethysmography (PPG) signals. Additionally, we compared radar-derived JVP signals with previously recorded catheterization data from a patient with early-stage heart failure to assess clinical relevance. Signal localization was successfully achieved within a direction-of-arrival (DoA) range of $-20^{\circ }$ to $+20^{\circ }$, demonstrating the radar’s precision. While the selected parameters consistently yielded good performance in our set-up, individual anatomical differences may require subject-specific calibration. These findings support the potential of 60 GHz FMCW radar for contactless JVP monitoring, with promising implications for early detection and remote management of heart failure.

## 1. Introduction

Heart failure (HF) occurs when the heart is unable to pump the required amount of blood adequately to meet the demands of the entire body. This condition can arise because of either the heart’s inability to fill with an adequate blood volume or a weakened capacity to pump effectively. HF can potentially impair the functionality of vital organs, such as the brain, liver and kidneys. In Australia, approximately 15% of hospitalizations have been attributed to HF [1]. The yearly count of HF-related hospital stays exceeds 179 000 in Australia alone, resulting in an estimated cost of more than $2.68 billion due to the necessity of inpatient care following readmissions [2]. This financial burden is expected to escalate with the aging Australian population, similar to the trends observed in many other countries worldwide.

HF, also known as congestive heart failure (CHF), results in impaired blood pumping and venous congestion due to blood backing up into systemic and pulmonary veins. Right-sided HF is particularly associated with elevated venous pressure and gradual symptom onset, emphasizing the importance of right ventricular assessment. The jugular venous pulse (JVP) serves as a key indicator, offering insights into venous pressure and right heart abnormalities such as atrial fibrillation or tricuspid valve disorders [3]. Evaluating the JVP waveform helps clinicians assess the severity of venous congestion and right heart failure.

**Figure 1.**
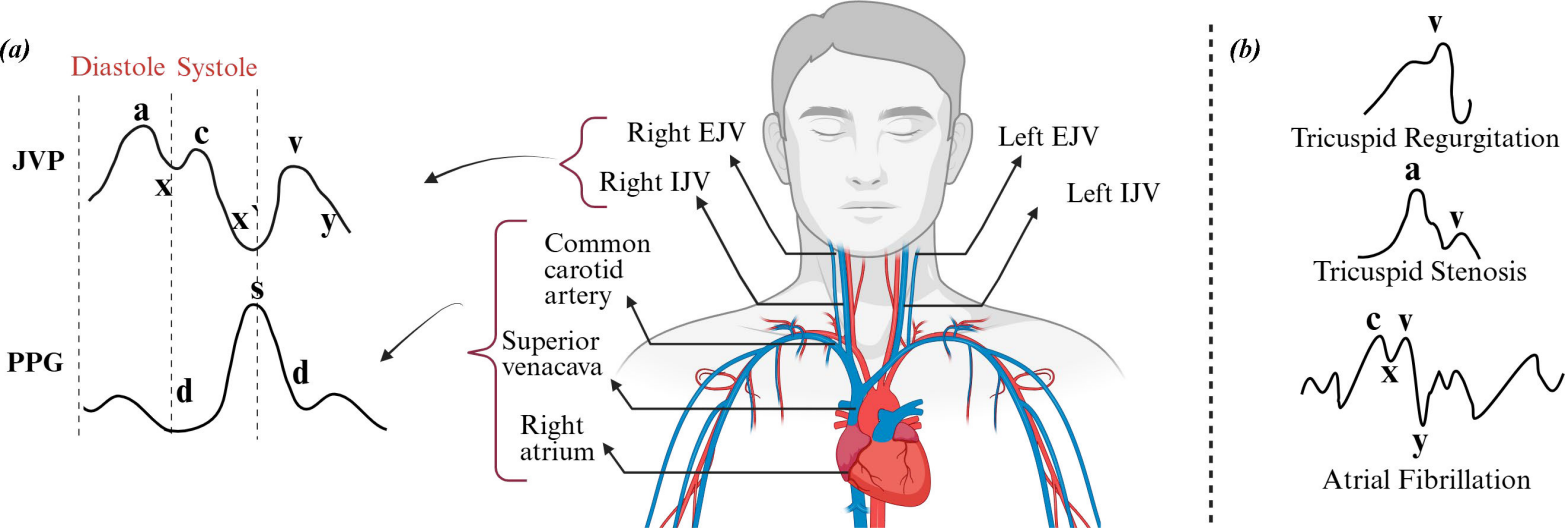
(*a*) Waveforms of the jugular venous pulse (JVP) and photoplethysmography (PPG) across a cardiac cycle are shown on the left, alongside a schematic of the superficial neck venous system connecting the internal jugular vein (IJV) and external jugular vein (EJV) to the right atrium. (*b*) Depiction of abnormal JVP patterns associated with various cardiac conditions.

As shown in figure 1*a*, the JVP waveform exhibits a biphasic pattern with distinct features: the a wave indicates atrial contraction, followed by the x descent (atrial relaxation), the c wave (ventricular contraction), a second x’ descent (tricuspid valve movement), the v wave (venous filling) and the y descent (atrial emptying). The neck contains three pairs of jugular veins: internal, external and anterior. The internal jugular vein (IJV) lies beneath the sternocleidomastoid muscle (SCM), while the external jugular vein (EJV) runs more superficially between the skin and the SCM. Since both JVP and photoplethysmography (PPG) signals are influenced by the same cardiac cycle, they exhibit notable phase alignments: the a wave precedes the PPG systolic peak, the v wave aligns with the dicrotic notch, and the y descent corresponds to the PPG’s falling phase [4]. These temporal correlations facilitate the analysis of systolic and diastolic features across both signals.

Figure 1*b* shows some abnormal JVP patterns that are commonly associated with various heart conditions. These abnormal patterns serve as important indicators of the underlying cardiovascular issues [5]. Therefore, analysing JVP waveforms can provide valuable insights into specific conditions, such as tricuspid regurgitation (TR), tricuspid stenosis and other forms of cardiac dysfunction [6].

Traditional JVP estimation is invasive and limited mainly to critically ill patients in intensive or coronary care settings because of the high risk of complications. Traditionally, the IJV has been preferred for JVP assessment due to concerns that valves in the EJV may interfere with accurate measurements. However, this view is outdated. The IJV also contains valves that do not hinder pressure evaluation and may even enhance the visibility of venous waveforms by amplifying atrial pulsations, as noted by Steven McGee [7]. While the IJV remains suitable for invasive procedures like catheterization, the EJV is better suited for non-invasive monitoring because of its superficial location, easier access and reduced interference from surrounding anatomy. Supporting this, studies [8,9] showed that EJV examination reliably detects elevated central venous pressure (CVP), highlighting its clinical value in non-invasive settings.

Non-invasive approaches for JVP estimation have explored PPG, though its reliability is often hindered by sensitivity to skin and tissue properties, which complicates calibration [4,10]. Radar-based radio-frequency (RF) sensing offers a more robust alternative for physiological monitoring, with previous studies employing 24 GHz microwave radar [11] and 900 MHz near-field radar [3] for JVP detection. However, the lower spatial resolution of 900 MHz systems and phase variation issues in near-field configurations limit their effectiveness in capturing detailed JVP waveforms.

This study, for the first time, employs a 60 GHz frequency-modulated continuous wave (FMCW) radar for non-contact JVP assessment. The higher operating frequency provides enhanced spatial resolution and sensitivity to subtle surface displacements. The Infineon BGT60TR13C radar module was selected for its integrated antenna-in-package design, reduced interference susceptibility, and flexible parameter configuration. Furthermore, this work is the first to utilize the Sim4Life platform from ZMT Zurich MedTech AG to investigate the impact of RF electromagnetic fields on the neck region, specifically in the context of high-frequency radar for JVP monitoring.

The key innovation lies in combining 60 GHz FMCW radar sensing with electromagnetic simulations to assess specific absorption rate (SAR) and power density (PD), supporting a safe and clinically applicable method for non-invasive JVP monitoring via the EJV.

## 2. Related work

### 2.1. Invasive method

The most common and widely available method for measuring JVP is cannulation, an invasive technique considered the gold standard. In this procedure, an experienced anaesthesiologist inserts venous catheters into the right internal jugular or subclavian vein (SVC). These catheters are then connected to a multi-modular monitor via a pressure transducer. After zeroing the transducer to the heart level, the mean venous pressure (mm Hg) is displayed in real-time on the monitor and recorded as invasive venous pressure [12].

Invasive methods can lead to various complications related to vascular access and typically require trained and skilled physicians.

### 2.2. Non-invasive contact method

Non-invasive techniques for JVP assessment are broadly categorized into intermittent and continuous methods. Continuous monitoring is particularly valuable for remote cardiac care. Several recent approaches employ pressure sensors, ultrasound, PPG and accelerometry.

Ultrasound imaging of the IJV is the most established method, offering real-time clinical insights [13–16]. However, its reliance on specialized equipment and trained personnel limits its practicality for long-term or ambulatory use [17].

To address these limitations, García-López *et al.* [4] proposed a reflectance-mode contact PPG system positioned at the upper thorax to capture the JVP waveform. Its single-point design offers potential for wearable integration but involves higher costs due to infrared camera requirements and demands precise placement for accurate readings. Complementing this, Proto *et al.* [10] introduced a cervical PPG device, placed on the neck as a more accessible and potentially cost-effective alternative for continuous monitoring, that employs capacitive strain gauge sensors to detect changes in the circumference of the neck, corresponding to the pulsations of the internal jugular vein.

Other modalities include accelerometer-based sensing, which captures skin vibrations linked to jugular and carotid flow [18]. While promising, this technique is susceptible to motion artefacts and less reliable in individuals with high body mass. Conroy *et al.* [3] explored a neck-worn near-field RF sensor placed over the IJV. Although more user-friendly, the device performance may be compromised by anatomical variability and movement.

In the broader context of PPG-based cardiovascular assessment, Elgendi *et al.* have made substantial contributions to the analysis of arterial pulse waveforms [19]. Although their work does not directly address JVP, it provides valuable recommendations for evaluating PPG-based algorithms and enriches the overall understanding of non-invasive cardiovascular sensing.

### 2.3. Non-invasive non-contact method

Non-invasive monitoring of the JVP has advanced through various contactless techniques. Amelard *et al.* [20] introduced a photoplethysmographic imaging (PPGI) system capable of capturing JVP waveforms without direct contact. While this method offers superior patient comfort in controlled settings, it necessitates stable patient positioning, limiting its applicability for long-term monitoring [3].

Further innovations include Tang *et al.*’s [21] sub-pixel image registration approach and Saiko *et al.*’s [22] specular reflection vascular imaging technique, both aiming to extract JVP waveforms non-invasively. Although promising, these methods require extensive validation across diverse populations.

Microwave radar technologies have also been explored. A prototype utilizing 24 GHz radar demonstrated the feasibility of JVP measurement; however, challenges in qualitative pulse interpretation persist [11]. Additionally, the subtle amplitude of JVP signals (typically, 6−8 cm H_2_O) [12] complicates detection, especially when using palpation-based methods that may compress the vein and obscure the waveform.

FMCW radar systems, at higher frequencies, have emerged as a viable solution for non-contact JVP monitoring. These systems can detect minute surface motions associated with cardiac activity, offering high-resolution data without direct skin contact [23–28]. Study also shows that integrating low-cost superstrates has enhanced near-field pulse sensing capabilities [29].

The adoption of FMCW radar technology holds promise for remote and continuous monitoring of heart failure patients, potentially reducing the need for frequent hospital visits and enabling timely clinical interventions. This study aims to validate the efficacy of a 60 GHz FMCW radar system for non-invasive JVP assessment, evaluating its performance through experimental data and exploring its potential for broader clinical application.

## 3. Material and methods

### 3.1. System design

**Figure 2.**
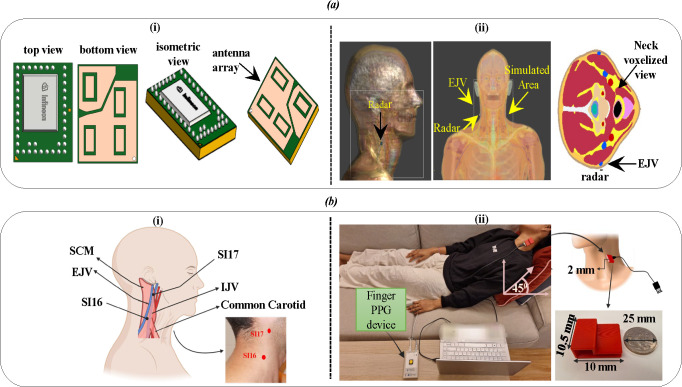
(*a*) Overview of FMCW radar simulation set-up. (i) 60 GHz radar system with integrated phased array antenna featuring 1 transmitter (Tx) and 3 receivers (Rx), (ii) radar positioning relative to the simulated area utilizing a realistic human model (Duke). (*b*)(i) Small intestine (SI16) and (SI17) acupoints near the SCM muscle serve as anatomical landmarks for radar alignment over the jugular vein. (ii) Experimental set-up with radar placed near SI16 and finger PPG attached to monitor cardiac activity.

This study utilizes a commercially available 60 GHz FMCW radar sensor (BGT60TR13C, Infineon Technologies AG) as shown in figure 2*a*(i), selected for its high sensitivity to sub-millimetre displacement and ability to provide range resolution [25–27]. The radar features one transmit and three orthogonally placed receive antennas within a compact 6.5×5.0×0.9 mm${,}^{3}$ package. Operating in the 58−63 GHz band, the device supports customization of parameters such as samples per chirp ($N_{\text{s}}$), chirps per frame ($N_{\text{c}}$) and chirp duration ($T_{\text{c}}$), with respective antenna gains of 6 and 10 dBi for transmit and receive channels. Data collected across the virtual antennas of the radar forms a three-dimensional cube, with fast and slow time information used for subsequent signal processing (see §S2 and electronic supplementary material, figure S2 for more details).

To assess electromagnetic interactions and safety, we employed the Sim4Life simulation platform (ZMT Zurich MedTech AG) [30], using the high-resolution Duke V3.0 voxel model. Figure 2*a*(ii) clearly shows that simulations targeted the neck region, where the EJV lies close to the surface, making it suitable for surface-level radar monitoring. Finite-difference time-domain (FDTD) methods [31,32] were used to evaluate specific absorption rate (SAR) and power density (PD) at various frequencies (900 MHz to 35 GHz), see electronic supplementary material, figures S3 and S4 in section S6. Also, the SAR and PD were calculated with a focus on 2 mm tissue depth. The simulation set-up confirms that higher-frequency signals like 60 GHz are well-suited for localized surface measurement.

Optimal radar placement is essential to ensure signal strength without saturation or loss of detail due to distance. To maximize physiological signal detection, we first identified the most suitable neck location for targeting the jugular vein. Given that venous return and pressure dynamics are influenced by gravity, the jugular pulse is best observed at a reclined angle between 30° and 45°.

To guide positioning, we referred to small intestine (SI) acupuncture points SI16 and SI17, as shown in figure 2*b*(i). SI16, located posterior to the sternocleidomastoid muscle [33], was identified as the optimal site for radar placement. A healthy adult female subject (aged 26−30) was positioned at angles of 0°, 45° and 90°, and both radar and finger PPG signals (sampled at 273.1 Hz) were collected for 30 s at each angle (figure 2*b*(ii)). The radar enclosure was 3D printed using bio-compatible plastic to facilitate precise positioning. Multiple parameter configurations were tested to optimize JVP detection.

To validate system sensitivity, we simulated JVP-like movement using a speaker plate facing the radar and emitting a synthetic beat signal composed of two frequencies ($f_{1}=50$ Hz and $f_{2}=54$ Hz):


(3.1) $$S_{\text{speaker}}(t)=a_{1}\cdot \cos(2\pi f_{1}t)+a_{2}\cdot \cos(2\pi f_{2}t)$$

**Figure 3.**
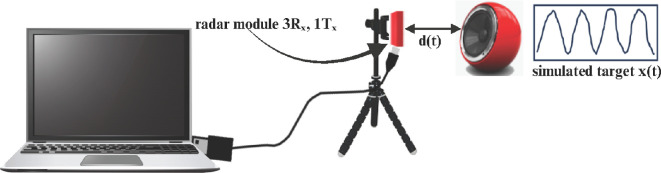
A speaker plate, positioned to face the radar, simulates a patient target in this experimental set-up. The experiment involves the synthesis of an audio signal, meticulously composed of two specific frequencies, 50 Hz and 54 Hz, to evaluate the radar system's response.

As shown in figure 3, this set-up emulates superficial pulsatile motion and serves as a controlled environment for algorithm evaluation [34].

This integrated material and system design approach enables both safety validation and signal fidelity assessment, laying the foundation for reliable non-contact JVP monitoring using high-frequency radar.

### 3.2. Methods

The workflow for the detection of the JVP is described in this section.

**Figure 4.**
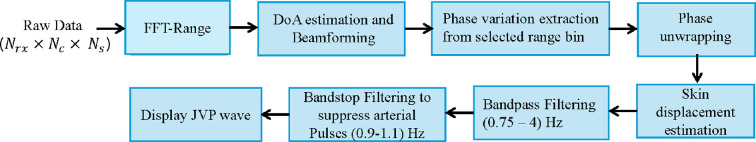
Flowchart of the data processing sequence. After reshaping the raw data, an FFT is performed on the samples to obtain the range information. Upon computation of FFT, displacement is calculated from the phase variation, which is then used to extract the JVP.

The data cube contains the normalized ADC samples of the received signal. Each frame of data consists of the number of receiving antennas ($N_{\text{rx}}$), the number of chirps per frame ($N_{\text{c}}$), and the number of samples per chirp ($N_{\text{s}}$). After obtaining the frame data of dimension ($N_{\text{rx}}=3,N_{\text{c}}=8,N_{\text{s}}=128$), sampled at a sampling rate of 1 MHz, is transformed into a complex range profile through the application of an Fast Fourier Transform (FFT) over the fast time dimension. Peaks within the FFT spectrum of the range indicate the presence of a target within the mmWave radar’s detection area. The overall data processing sequence is shown in figure 4.

Below, we detail each step in the signal processing sequence, accompanied by the corresponding pseudo-codes.

#### 3.2.1. FFT analysis and IQ complex signal extraction

— The raw data vector is first reshaped into a matrix.
— FFT processing was applied to every chirp pulse in each frame and to each antenna. (The optional windowing technique can be used before the FFT for spectrum interpolation.)
— The FFT outputs are averaged to reduce the noise effect for each antenna signal and from each frame.
— From the averaged spectrum of the raw data, each antenna detected the first spike as a radar echo from the patient’s skin.
— The inverse fourier transform (IFFT) of first spectrum bin to extract IQ complex signal for each antenna.

The FFT is performed separately for the best two antennas (1 and 3) in terms of their signal-to-noise ratio (SNR). Additionally, the averaging process is crucial here for noise reduction and signal clarity enhancement.

**Figure:**
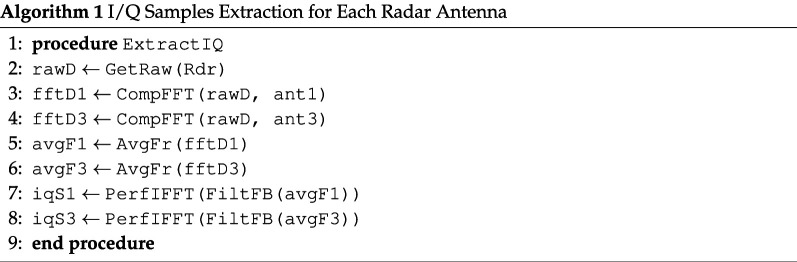


#### 3.2.2. Beamforming to enhance signal-to-noise ratio

While the I/Q extraction process follows the approach in [26], our method diverges in beamforming. The eigenspace-based beamforming method (ESBM) used for blood pressure monitoring in [26] proved unstable in our setting due to ill-conditioned covariance matrices. To address this, we adopt eigen-beamforming—an adaptive method that leverages the dominant eigenstructure of the signal to optimize antenna weighting for enhanced stability and SNR.

Eigen-beamforming is widely used in radar and communication systems for directionally selective reception [35]. It relies on eigenvalue decomposition of the signal covariance matrix to identify the principal signal direction while suppressing noise and interference. For two-antenna configurations, the signal subspace is defined by the eigenvector with the highest eigenvalue [36], enabling coherent signal combination and effective noise mitigation.

**Figure:**
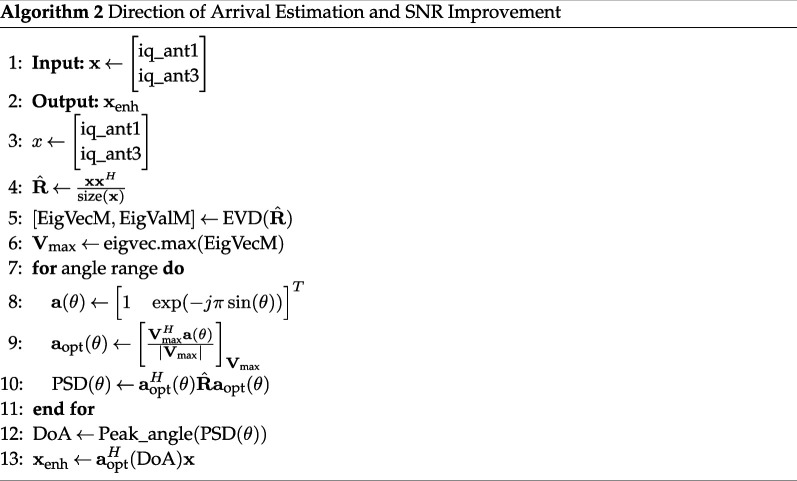


Selecting this modified version of beamforming is due to one bin selection from the FFT-range function, which induces pure sine wave function as an I/Q signal. In such one bin output signal, very low noise contribution is observed in the IQ signal, which implies an almost noise-free observation vector x. So, the covariance matrix


$$\hat{R}=\frac{xx^{H}}{\text{size}(x)}$$

is by definition a rank-one matrix, and with almost noise-free observation, this covariance matrix is ill-conditioned (the ratio of the highest eigenvalue to the smallest eigenvalue is much greater than 1) and hence non-invertible. Constructing the steering vector (vector of signal phases in each receiving antenna) for a given direction of Arrival α:


(3.2) $$A(\alpha )=\left[\begin{array}{c} 1\\ \exp(-i\pi \;\sin(\alpha )) \end{array}\right]\;.$$

The received signal power at the output of the beamformer is calculated with respect to the angle α:


(3.3) $$\text{PSD}(\alpha )=A_{\text{proj}}{\hat{R}}_{X}A_{\text{proj}}^{H}\;,$$

such that:


(3.4) $$A_{\text{proj}}=AA^{H}A(\alpha )\;.$$

This last expression is a A(α) projection on the signal subspace generated by the eigenvector A associated with the highest eigenvalue. The objective of this projection is to reduce noise and other radar reflections.

To address the noisy circular pattern in the I/Q plane, Taubin circular regression with a singular value decomposition (SVD) based implementation was employed to correct offset and noise. See electronic supplementary material, figure S6 in section S6. This method was selected for its robustness and reduced algebraic error, offering greater accuracy than traditional least squares approaches [26,37].

#### 3.2.3. Skin displacement extraction from phase variation

To extract the phase variation of reflected radar echoes caused by skin displacement, we extract the first instantaneous phase of the output signal from our eigen-beamformer. This instantaneous phase must be unwrapped to retrieve its linearly growing nature over time. Only phase fast variation, rather than linear behaviour of the instantaneous phase, carries information about the jugular pulse. To extract these variations, we use phase differences between successive phase samples.


$$d_{\text{skin }i_{\text{Frame}}}={\hat{\theta }}_{\text{skin }i_{\text{Frame}}}\times \frac{\lambda }{4\pi }$$

Skin displacement is related to phase variation using wave number $k=\frac{2\pi }{\lambda }$ rad m^−1^, which represents the phase variation of EM waves over space. The factor $\frac{1}{2}$ is due to the round-trip time needed for the radar signal to hit the skin and come back to its receiver.

**Figure:**
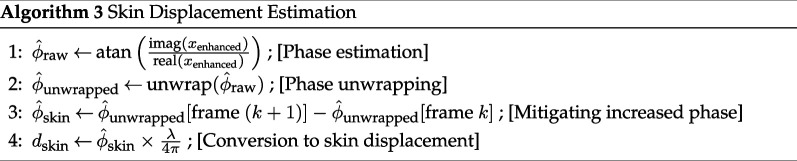


Studies have reported that the visibility of most of the JVP components falls below 4 Hz [21,38]. Therefore, a bandpass filter (0.75−4 Hz) was applied to the displacement signal, followed by a bandstop filter (0.9−1.1 Hz) to reduce arterial pulsation impact. While the filtering process was guided by a correlation threshold related to the viability of pulse waves, the filter was chosen carefully to trade off between reducing the arterial signal interference and preserving the components of the JVP waveform. We select the best pulse from the recording as a reference to detect the best JVP pulses. We scan all recordings for pulses with the highest correlation coefficient using correlation. When we detect the desired number of best JVP pulses, we mark their peak values. We can choose the degree of resemblance concerning the reference pulse by fixing the correlation threshold of accepted JVP pulses. The JVP signal presents a transient effect on both ends of the recording because the filter time delay line is not fully loaded. The length of these transient portions of the signal is equal to the combined group delay of 765 samples caused by both the bandpass filter and bandstop filter.

## 4. Results

In this section, we present a detailed discussion of the simulation outcomes along with the data analysis results. The data analysis section covered both time and frequency domain analyses at various inclinations, along with a comparison between the JVP and the PPG signal. Additionally, we compared the spectrum of the non-invasive JVP measurements with data obtained from the right heart catheterization (RHC) method.

### 4.1. Simulation analysis result

The SAR and PD reduce with the increase in distance for high frequencies. In the design operation of our device, we have carefully maintained a 2 mm distance between the radar and the skin surface. This specific distance has been chosen to ensure that the SAR and PD values are kept within safe and acceptable limits [39]. At this 2 mm distance, our measurements indicate that the SAR is approximately 1.01 W kg^−1^, and the PD is 0.002 W m^−2^. For reference, see electronic supplementary material, figure S7 in section S6. These values are well within the regulatory safety standards established by the Federal Communications Commission (FCC) [40] and the International Commission on Non-Ionizing Radiation Protection (ICNIRP) [41], ensuring that the device operates effectively while minimizing any potential thermal effects on the tissue. The safety guidelines are discussed in the electronic supplementary material, section S3.

### 4.2. Data analysis result

For our experiment, the selection of antennas was guided by the SNR criterion. This SNR metric, which correlates to the amount of reflected energy captured, is influenced by the positioning of the antennas relative to the vein. The optimal antenna configuration is the one that positions the vein equidistantly between the selected pair of antennas. From the FFT analysis, the first FFT-range bin was chosen as the logical reference point, corresponding to the initial radar reflection from the closest obstacle, typically the skin. The other reflections (i.e. bins) relate to the other obstacles present in the environment. For reference, see electronic supplementary material, figure S8 in section S6.

**Figure 5.**
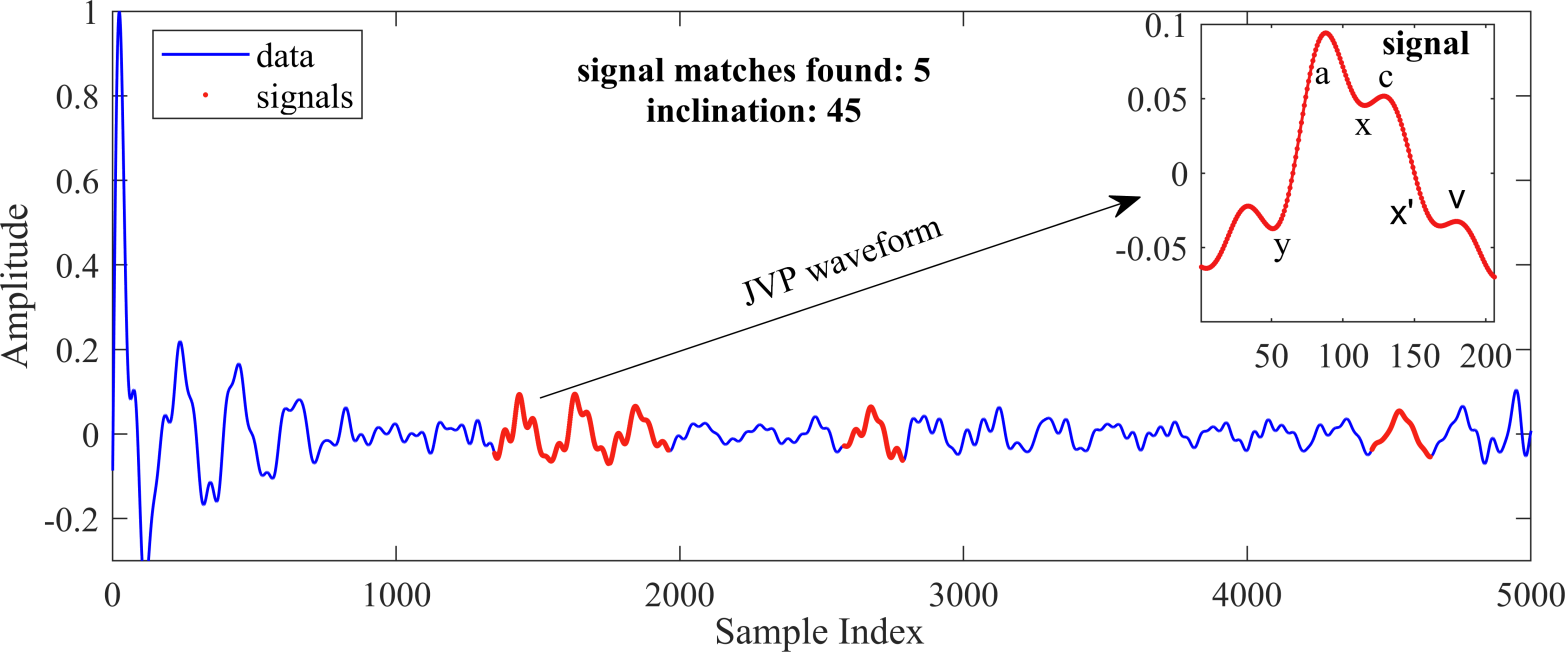
Detected JVP waveforms (red) overlaid on the full signal (blue); inset zooms into a JVP waveform marked by points indicating the ascent and descent of the waveform, respectively.

Our study explores a proof-of-concept technology for JVP detection using varying radar parameters, such as samples/chirp, chirps/frame and sampling frequency. Our findings, illustrated in figure 5, indicate improved JVP visibility with higher samples/chirps; here, we used 128 samples/chirp, 8 chirps/frame and a 1000 kHz sampling frequency. The chirp duration was 132.987 μs.

#### 4.2.1. Time and frequency domain analysis at different angle orientations

The quality of the JVP signal changes with the body’s inclination, and this phenomenon is physiological. In a healthy person, the visible jugular veins are fully collapsed when standing and are often distended to a variable degree when the person is supine. Selecting an appropriate intermediate position permits the top of the column to become visible in the neck between the clavicle and the mandible. So, as the angle increases, the carotid pulse is expected to be more dominant compared with JVP, overshadowing its features.

**Figure 6.**
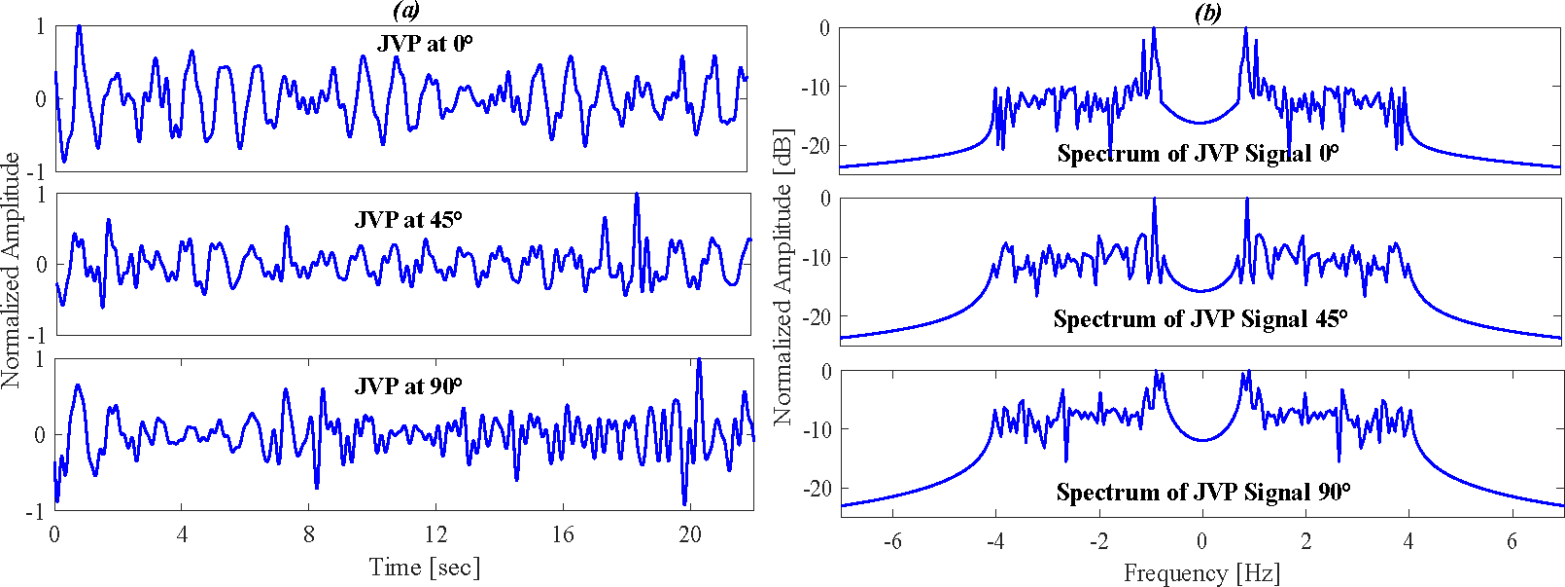
Analyses of JVP signal at both time (*a*) and (*b*) frequency domain for different angle orientations of 0, 45 and 90°.

Figure 6*a* illustrates the JVP signals in the time domain, and figure 6*b* shows the frequency spectrum of JVP signals at three different angles of inclination: 0°, 45° and 90°. Body position significantly affects JVP visibility. Therefore, the angle of accuracy plays a crucial role in measuring JVP accurately. At 0°, increased venous return enhances carotid pulsation, which may mask the JVP due to its stronger amplitude. This is evident in figure 6*b*, where higher signal amplitudes and spectral peaks near 0 Hz reflect slow venous flow under gravity. At 45°, spectral peaks shift and amplitudes stabilize, allowing clearer identification of JVP pulses as gravitational effects are balanced. By contrast, at 90°, gravity reduces venous return, attenuating the JVP signal and complicating interpretation. Thus, 45° is deemed the optimal angle for reliable JVP assessment.

#### 4.2.2. Comparison between JVP and PPG signal

**Figure 7.**
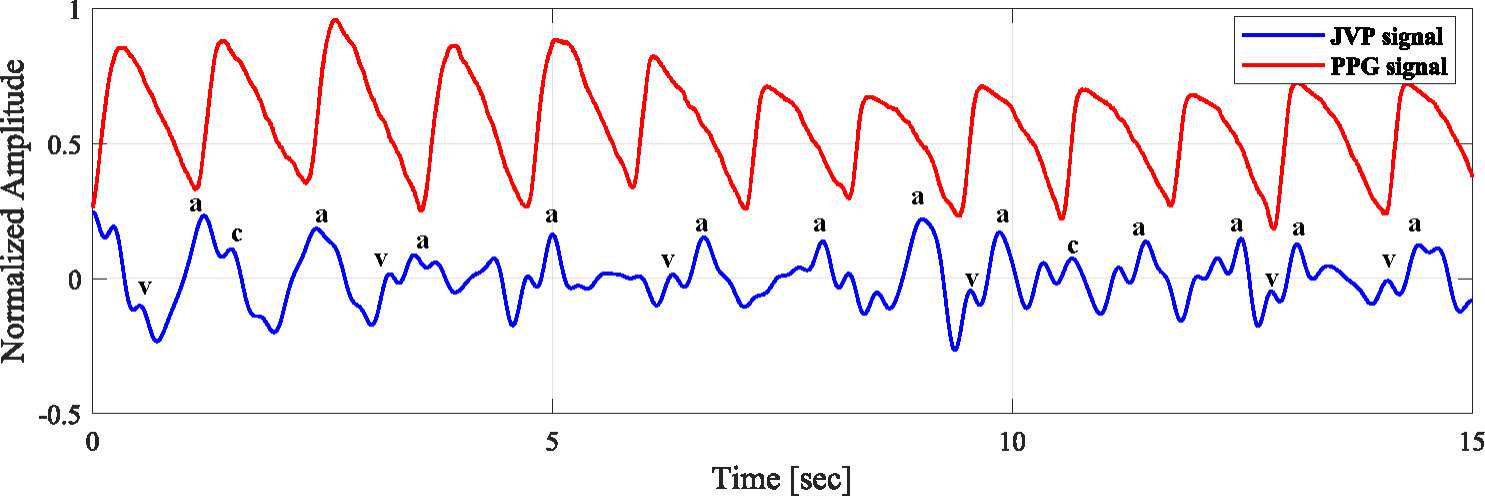
Comparison of JVP and PPG signals from synchronized measurements, highlighting temporal alignment and amplitude differences between JVP and PPG pulsations.

Figure 7 shows the representation of the subject’s JVP signal and PPG signal. PPG was recorded simultaneously from the right index finger using the mentioned device while the JVP was recorded from the neck at an inclination. We have implemented a synchronized measurement approach to ensure that the alignment process is both transparent and replicable. This is a synchronization based on the correlation of common frequency between two signals. As both JVP and PPG exhibit cardiac cycles, they are influenced by heart activity. The most common frequency range where PPG and JVP overlap is 0.8 Hz to 2 Hz. In this experiment, the common frequency is 1.13 Hz. From figure 7, we can see that the rising peaks of PPG parallel the descents of the JVP. However, it is to be noted here that this alignment is not consistent across all cardiac cycles, as the respiratory cycle may influence breathing, blood flow, venous return timing, physiological condition, etc.

#### 4.2.3. Spectrum comparison of the non-invasive JVP and catheterized data

As a proof-of-concept feasibility study, the experiment was ethically limited to a healthy subject. While cardiac catheterization remains the gold standard for JVP assessment, it is invasive and typically reserved for critically ill patients. To provide comparative insight, we analysed RHC data from a patient with TR—an early marker of CHF, and compared its spectral characteristics with the JVP signal from our healthy subject. This comparison highlights waveform differences between normal and early-stage CHF conditions.

**Figure 8.**
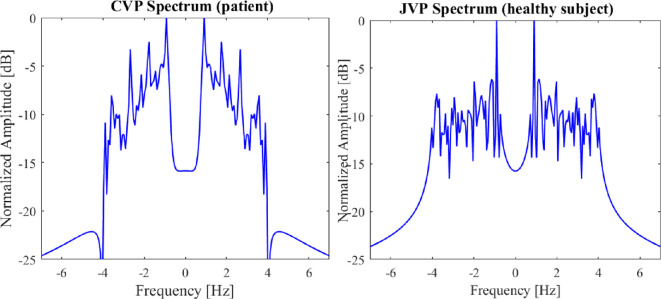
Frequency spectrum comparison of RHC CVP signal and sensor recorded JVP signal. Both spectra are shown in normalized amplitude (dB) over a frequency range, emphasizing the frequency components and amplitudes that are characteristic of cardiovascular waveforms.

In figure 8, the broader spread and higher amplitude peaks in the CVP spectrum indicate turbulent and irregular blood flow, which is characteristic of TR. The spectrum of healthy subjects is characterized by narrower frequency bands and lower overall amplitude, indicating a more stable and regular venous pressure pattern with proper functioning of the tricuspid valve. The dominant frequencies in both spectra are related to the low-frequency components, which suggests a clinically significant correlation between them.

Of note, CVP is an invasive measure, and JVP is a non-invasive measure that reflects the right atrial pressure. Due to the non-invasive nature, there will be timing delays and attenuation because of the distance between the jugular vein and the right atrium. Also, JVP can be influenced by factors like the patient’s position, neck angle and even breathing pattern, which might not directly affect CVP. Nonetheless, the correlation between them confirms that JVP can serve as a useful non-invasive estimate for understanding right heart health.

## 5. Discussion

This study demonstrates the feasibility of using a 60 GHz FMCW radar for non-invasive JVP extraction. The radar’s high resolution, integrated antenna design and sensitivity to subtle skin displacements make it well-suited for detecting venous pulsations. All recordings utilized the maximum bandwidth of 5.5 GHz in close-range measurements.

JVP signal quality was found to depend on radar–subject distance. Too close a placement caused overlap between the initial reflection and the DC component in the FFT spectrum, while excessive distance diminished signal amplitude. An optimal spacing of approximately 2 mm was maintained. Additionally, inter-bin mixing effects and hardware constraints—particularly the tight coupling between sampling frequency and chirp configuration—necessitated precise tuning of system parameters. At least eight chirps were required to reliably extract phase information due to the radar’s fast chirp generation characteristics. These findings highlight the potential of high-frequency radar for integration into non-contact clinical monitoring systems, offering a promising alternative to invasive techniques for early cardiovascular assessment.

Through meticulous evaluation, we established the subsequent configuration for JVP estimation with this radar:

— Higher sampling rate for a fast time (higher number of samples per chirp) to increase FFT-range resolution. Because a lower value implies a reduction of the signal quality and even burying the signal in noise.
— Suppress averaging on FFT-range spectrums over all chirps to avoid losing JVP signal details.
— Higher frame rate to get more time resolution for JVP signal.
— In some situations, a signal with low SNR in one antenna can significantly reduce the performance of the beamformer and, eventually, the pulse detection. Therefore, an antenna selection procedure must be adopted.

Also, a shorter chirp duration is helpful because if the chirp is longer, the chirp reflection collides with the transmitted one. Therefore, for better results, we narrowed down the parameter values in table 1. Longer chirp durations are permissible if the radar-skin distance increases. Additionally, increasing the receiver’s gain is essential under these conditions.

**Table 1.** Radar configuration for optimal JVP estimation.

parameter	value/condition
number of samples per chirp	128 or the highest possible value
frame rate	250 Hz or the closest possible value
number of chirps per frame	≥8 chirps
chirp duration	0.00013 s or closest possible value

The receiver chip employs only an in-phase (I) mixer, producing a real-valued intermediate frequency (IF) signal without a quadrature (Q) component. Applying a Fourier transform yields complex I/Q data, enabling phase analysis across frequencies. However, this architecture is susceptible to signal fading caused by phase offsets between the reference and reflected signals, limiting its temporal accuracy compared with true I/Q radar systems. Through the configuration illustrated in figure 3, we establish the critical enhancements offered by quadrature (Q) radar receivers over non-quadrature or in-plain (I) counterparts, as outlined in [42].

The original beat signal exhibits a pronounced spike thanks to its high amplitude. By contrast, radar-based beat reconstruction reveals an inconsistent envelope signal stemming from the phase difference between the received signal and the local oscillator. This experiment not only confirms the effectiveness of our algorithm but also sheds light on the constraints related to the hardware (see electronic supplementary material S6, figure S9). Therefore, we prefer to use a sensor equipped with an IQ mixer for future experiments.

JVP signals are often obscured by dominant arterial or respiratory components, complicating their isolation and analysis. While Suzuki *et al*. [11] investigated non-contact JVP monitoring using 24 GHz radar, it did not address arterial interference. To mitigate this, we applied a supplementary bandstop filter to suppress arterial pulsations. Additionally, DoA estimation helped accurately localize the JVP signal. Inspired by the application of 60 GHz radar for blood pressure estimation in [26], we employed a radar to explore and analyse JVP as a proof of concept. Our study demonstrated the radar’s potential in JVP estimation, overcoming its design limitations.

### 5.1. Potential challenges and opportunities

While this work provides insight into the feasibility of a contactless and non-invasive JVP measurement, there are some potential challenges and limitations to consider. This proof-of-concept study needs to follow the usual clinical validation pathway for medical devices and technology, in which a control and target cohort will be recruited and studied against the gold standard (baseline).

### 5.2. Future work

Our ongoing efforts aim to clinically gather evidence of the system’s efficacy with target and control cohorts of participants within a clinical setting through extensive analysis and validation. This study occurs before a proper clinical trial, which can be relatively quick due to this technology’s potentially favourable regulatory classification because of its non-invasive, contactless and low-risk features. It is also required to enhance the radar system’s signal-to-noise performance and explore the integration of automated signal-processing techniques. Also, we plan to incorporate wavelet-based techniques [43] in our future signal enhancement pipeline to further improve robustness under noisy conditions.

## 6. Conclusion

This study demonstrates the feasibility of using a 60 GHz FMCW radar for non-invasive JVP estimation, offering a compact and cost-effective alternative to conventional methods. We conducted simulation analyses across multiple frequencies and identified 60 GHz as the most suitable choice for this study. Despite hardware constraints, we successfully identified key signal parameters and validated radar-derived JVP signals through comparison with finger PPG data. A spectral comparison between the estimated JVP signal and catheter-acquired CVP data has also been presented. We also examined the influence of body posture and anatomical variability on signal quality. These findings highlight the radar’s clinical potential, particularly for early heart failure detection in remote settings. Future work will focus on larger cohorts and refining accuracy across diverse physiological conditions.

## Data Availability

The approved ethics prevented us from the public release of unidentified data and at this stage only allowed limited research access. The simulation results of the jugular vein using Sim4Life can be found via Figshare [44], and the GitHub repository for this work can be accessed [45]. Supplementary material is available online [46].
